# Stem Cell-Derived Exosomes Protect Astrocyte Cultures From *in vitro* Ischemia and Decrease Injury as Post-stroke Intravenous Therapy

**DOI:** 10.3389/fncel.2019.00394

**Published:** 2019-09-03

**Authors:** Xiaoyun Sun, Ji-Hye Jung, Oiva Arvola, Michelle R. Santoso, Rona G. Giffard, Phillip C. Yang, Creed M. Stary

**Affiliations:** ^1^Department of Anesthesiology, Perioperative and Pain Medicine, Stanford University School of Medicine, Stanford, CA, United States; ^2^Division of Cardiovascular Medicine, Department of Medicine, Stanford University School of Medicine, Stanford, CA, United States; ^3^Stanford Cardiovascular Institute, Stanford University School of Medicine, Stanford, CA, United States

**Keywords:** glia, iCM, iPSC, pluripotent, MCAO, cerebral ischemia

## Abstract

In the present study, we assessed efficacy of exosomes harvested from human and mouse stem cell cultures in protection of mouse primary astrocyte and neuronal cell cultures following *in vitro* ischemia, and against ischemic stroke *in vivo*. Cell media was collected from primary mouse neural stem cell (NSC) cultures or from human induced pluripotent stem cell-derived cardiomyocyte (iCM) cultures. Exosomes were extracted and purified by polyethylene glycol complexing and centrifugation, and exosome size and concentration were determined with a NanoSite^TM^ particle analyzer. Exosomes were applied to primary mouse cortical astrocyte or neuronal cultures prior to, and/or during, combined oxygen-glucose deprivation (OGD) injury. Cell death was assessed *via* lactate dehydrogenase (LHD) and propidium iodide staining 24 h after injury. NSC-derived exosomes afforded marked protection to astrocytes following OGD. A more modest (but significant) level of protection was observed with human iCM-derived exosomes applied to astrocytes, and with NSC-derived exosomes applied to primary neuronal cultures. In subsequent experiments, NSC-derived exosomes were injected intravenously into adult male mice 2 h after transient (1 h) middle cerebral artery occlusion (MCAO). Gross motor function was assessed 1 day after reperfusion and infarct volume was assessed 4 days after reperfusion. Mice treated post-stroke with intravenous NSC-derived exosomes exhibited significantly reduced infarct volumes. Together, these results suggest that exosomes isolated from mouse NSCs provide neuroprotection against experimental stroke possibly *via* preservation of astrocyte function. Intravenous NSC-derived exosome treatment may therefore provide a novel clinical adjuvant for stroke in the immediate post-injury period.

## Introduction

Stroke remains the second-leading cause of death worldwide, with ischemic/embolic stroke accounting for ∼87% of total stroke occurrence (Benjamin et al., 2019). To date, early restoration of cerebral blood flow with thrombolytics remains the only effective intervention to minimize the evolution of injury. The failure to develop novel adjuvant therapies may lie in a historical focus on genes and cell-signaling pathways that are predominant in terminally differentiated cells. Neural progenitor cells persist in the adult brain and recent evidence (Peron and Berninger, 2015) describes latent neurogenic pathways re-activated after ischemic injury that act to preserve and restore neuronal function. Stem-cell targeted therapies have more recently been studied intensely as a therapeutic option to translate the developmental capacity of embryogenesis to treat a wide array of adult diseases and injuries. However, early promising results with directly transplanted stem cell lines (Hao et al., 2014) have been offset by immune-rejection, and tumorigenesis (Solter, 2006; Kawai et al., 2010). Most recently, in the setting of stroke therapy, the beneficial outcomes of stem cell therapy have been localized to their paracrine effects, and not mediated by cell replacement or transplanted cell differentiation (Chen et al., 2014; Xin et al., 2014). Therefore, a more immediate focus has been delineating the cellular mechanisms of the stem cell *secretome*.

Exosomes are nanoscale vesicles (≈30–130 nm in diameter) released by most cell types after multivesicular bodies (MVBs) fuse with the plasma membrane (Colombo et al., 2014). Exosomes are present in the circulation and contain a variety of proteins, nucleic acids and lipids derived from their host cells, facilitating intercellular communication and regulating recipient cell function (Robbins et al., 2016). Exosomes can cross the blood-brain barrier (BBB), and are transported into brain cells *via* pinocytosis, direct fusion with the plasma membrane, or binding to cell surface proteins and cell adhesion molecules (Colombo et al., 2014). Recent work utilizing exosomes for ischemic stroke therapy have attributed improved functional outcome to their cargoes, which includes microRNAs, DNAs, lipids, proteins and RNAs (Chen and Chopp, 2018). Deng et al. (2018) recently demonstrated that human neural stem cell (NSC)-derived exosomes had the best effect on neuronal recovery after *in vitro* ischemia compared to exosomes derived from terminally differentiated neurons or glia. These observations suggest that application of NSC-derived exosomes may be a promising new avenue for stroke therapy, however, their cellular targets remains poorly defined. Specifically, whether there are species-, organ- or cell-type specific effects of stem cell-derived exosomes on neuroprotection remains a critical gap impeding therapeutic implementation.

Notably, re-activation of latent embryonic pathways in the brain following ischemic injury have been shown to be regulated by astrocytes (Gotz et al., 2015), specialized glia critical in maintaining neuronal homeostasis in health and in response to injury (Clarke and Barres, 2013). Our lab and others have demonstrated that preserving astrocyte survival and function can translate to neuronal protection and reduced injury (Ouyang et al., 2013; Stary and Giffard, 2015), further supporting their potential as therapeutic targets for stroke. Therefore, in the present study, we assessed the effect of exosomes derived from mouse NSC cultures on primary mouse astrocyte and neuronal cultures subjected to *in vitro* ischemic injury. To assess inter-species exosome protection we also assessed the effect of exosomes derived from human induced pluripotent stem cell-derived cardiomyocytes (iCM) on primary mouse astrocyte cultures subjected to *in vitro* ischemic injury. After observing marked protection primarily by mouse NSC-derived exosomes in primary mouse astrocyte cultures, we then assessed their efficacy with post-injury intravenous application in an *in vivo* model of ischemic stroke.

## Materials and Methods

### Primary Mouse Neural Stem Cell Cultures

**FIGURE 1:**
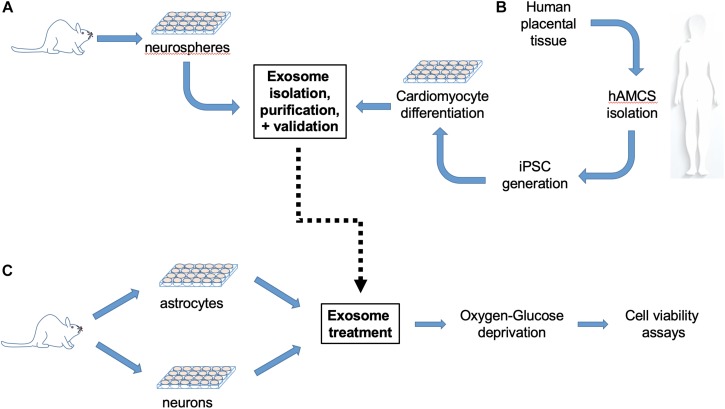
Experimental workflow. **(A)** Mouse neural stem cells (NPCs) isolated from newborn mice were cultured for 2–3 days until proliferation to neurospheres, and then processed for exosome extraction, purification and size validation. **(B)** In parallel, exosomes were isolated from cultured human induced pluripotent stem cell (iPSC)-derived cardiomyocytes (iCMs) generated from human amniotic mesenchymal stem cells (hAMSCs). **(C)** Primary cerebral cortical astrocyte cultures were prepared from post-natal day 1–3 mice, plated for 21 days and then subjected to 6 h combined oxygen/glucose deprivation (OGD) with or without exosome treatment, followed by 24 h reperfusion prior cell viability assay. Neuronal cultures prepared from the cortices of embryonic day 15–16 mouse fetuses were plated for 12–13 days and then subjected to 70 min OGD with or without exosome treatment, followed by 24 h reperfusion prior to cell viability assay.

All animal experiments were approved by Stanford University Animal Care and Use Committee (Stanford, CA, United States) and conducted according to the National Institutes of Health guidelines for animal welfare. Mouse NSCs were isolated from newborn mice within 24 h as previously described (Sun et al., 2015) (Figure 1). In brief, brains were removed, freed of meninges, and diced with a sterile razor blade in dissociation buffer [DMEM (Thermo Fisher Scientific, Waltham, MA, United States) containing 2.5 U/ml papain, 250 U/ml DNase I (Thermo Fisher Scientific), and 1 U/ml Dispase II (Roche Diagnostics, Indianapolis, IN, United States)]. After 1 h incubation the cells were washed three times with DMEM plus 10% fetal bovine serum (FBS, HyClone, Logan, UT, United States). The cells were then resuspended in plating medium, Neurobasal A (Thermo Fisher Scientific), with 2 mM L-glutamine, 100 U/ml penicillin, and 100 ug/ml streptomycin (Thermo Fisher Scientific), B-27 without vitamin A (Thermo Fisher Scientific), 20 ng/ml fibroblast growth factor-2 (Peprotech, Rocky Hill, NJ, United States), and 20 ng/ml epidermal growth factor (Peprotech), and plated at a density of one brain per six-well plate. Neural precursor cells proliferated and started to form neurosphere morphology (NSCs) in 2–3 days.

### Human Induced Pluripotent Stem Cell-Derived Cardiomyocytes

Human induced pluripotent stem cell (iPSC)-derived cardiomyocytes (iCMs) were generated from human amniotic mesenchymal stem cells (hAMSCs), as previously described (Ge et al., 2012) (Figure 1). In brief, human placental tissue was obtained from healthy subjects at the Stanford Healthcare Center. All donors provided written informed consent before collection. Placental tissue underwent serial enzymatic digestion with trypsin-EDTA (Thermo Fisher Scientific) in phosphate-buffered saline and HBSS (Thermo Fisher Scientific) containing 50 mg type I collagenase (Thermo Fisher Scientific), 0.01% papain (Sigma, St. Louis, MO, United States), and 10% FBS (HyClone). After digestion, the suspension was filtered and the cells were collected by centrifugation at 200 g for 5 min. hAMSCs were seeded in 6-well plates and infected with 150 μL of pHAGE2-EF1α-OKSM (courtesy of Mostoslavsky, G, Ph.D., Boston University) virus in the presence of polybrene (10 mg/mL; Sigma). On day 6 post-infection, the cells were trypsinized (trypLE; Thermo Fisher Scientific), and pre-seeded with irradiated mouse embryonic fibroblasts (MEF-irr; GlobalStem). The resulting iPSCs were identified and verified as spontaneous colonies forming in human embryonic stem cell (hESC) culture media. iPSCs were then differentiated toward cardiac lineage with small molecules as previously described (Tachibana et al., 2017).

### Exosome Extraction and Characterization

Exosome extraction was performed using a modified protocol by Chen et al. (2013). Briefly, Cell culture supernatant was centrifuged at 300 g for 10 min and passed through 0.2 um pore bottle top filter (BD Falcon, #352340, Thermo Fisher Scientific). One quarter volume of PEG (polyethylene glycol 8000, Thermo Fisher Scientific) stock solution [(20 g PEG8000 in 20 ml 0.5 NaCl phosphate-buffered saline (PBS, Sigma)] was added, then the total volume was brought to 50 ml with deionized H_2_O and incubated overnight at 4°C. Samples were then centrifuged at 1500 g for 30 min, then at 300 g for 5 min after removal of most supernatant. The final pellet was then resuspended in PBS. Protein concentrations were determined *via* the bicinchoninic acid method (BCA Protein Assay Kit; Pierce, Rockford, IL, United States). The Malvern Panalytical NanoSight^TM^ instrument (which utilizes Nanoparticle Tracking Analysis to characterize nanoparticles from 10 to 2000 nm) was used to define exosome size and concentration.

### Primary Mouse Astrocyte Cultures and Neuronal Cultures

Primary cerebral cortical astrocyte cultures were prepared from postnatal days 1–3 Swiss Webster mice (Charles River, Wilmington, MA, United States) as previously described (Dugan et al., 1995). Briefly, cortices freed of meninges were incubated in 0.05% trypsin/EDTA (Thermo Fisher Scientific) for 30 min at 37°C, mechanically dissociated, and plated in Falcon Primaria 24-well plates (Becton Dickinson, Lincoln, IL, United States) at 1–2 hemispheres per plate, in Eagle’s minimal essential medium, high glucose (Glutamic, Thermo Fisher Scientific) supplemented with 10% fetal bovine serum, (Hyclone, Logan, UT, United States), 100 u/ml penicillin, 100 ug/ml streptomycin (Thermo Fisher Scientific). Cultures were maintained at 37°C in 5% CO_2_, and medium was changed every 2 days before cell confluence, then twice/week after confluence, and utilized at day-*in-vitro* (DIV) 21. Relatively pure neuronal cultures, containing <1% astrocytes (Dugan et al., 1995), were prepared from the cortices of embryonic day (ED) 15 or 16 Swiss Webster mice. Cortices collected in ice-cold Eagle’s minimal essential medium (Thermo Fisher Scientific) were digested with 0.05% trypsin/EDTA for 15 min at 37°C, triturated, then plated in medium containing 5% FBS and 5% ES (Hyclone). The culture medium was replaced with glial-conditioned medium containing 5% ES and 2% B-27 (Thermo Fisher Scientific). Cytosine arabinoside (3 mol/liter, Sigma) was added 24 h after plating to inhibit glial proliferation. Cultures were used after 12–13 days (DIV 12,13) for oxygen-glucose deprivation (OGD) experiments.

### Cell Culture Injury and Exosome Treatment

Combined OGD was employed as *in vitro* ischemic injury as we have previously done (Voloboueva et al., 2008). Briefly, primary astrocyte and neuronal cultures were transferred to an anaerobic chamber (Coy Laboratory Products Inc., Grass Lake, MI, United States) with atmosphere gas partial pressure maintained of 5% CO_2_, 5% H_2_, and 90% N_2_. The culture medium was replaced by three washes with deoxygenated, glucose-free balanced salt solution (BSS0), pH 7.4, containing phenol red (10 mg/liter) and (in mM) NaCl 116, CaCl_2_ 1.8, MgSO_4_ 0.8, KCl 5.4, NaH_2_PO_4_ 1, NaHCO_3_ 14.7, HEPES 10. BSS5.5 contains 25 mM glucose in BSS0 and is the medium used for wash controls. Cultures were kept at 37°C in the anaerobic chamber for 6 h for astrocyte cultures (Sun et al., 2013) and 70 min for neuronal cultures (Wang et al., 2004). Oxygen tension was monitored with an oxygen electrode (Microelectrodes, Bedford, NH, United States) and was kept under 0.02%. OGD was terminated by adding glucose to the culture medium to a final concentration of 5.5 mM and returning the cultures to the normoxic incubator for 24 h prior to assessment of cell death. In separate (unpublished) results, we have observed protection by iPSC-derived exosomes in cardiomyocytes at doses of at 10, 25, 50, 100, 200, 500 μg with an optimal dose at 50 μg. In the present study, we assessed doses *in vitro* of 30, 60, and 90 μg.

### Cell Viability Assays

Extracellular release of lactate dehydrogenase (LHD) was measured to assess the extent of cell death as we routinely perform (Stary et al., 2017). LDH is an intracellular enzyme that catalyzes the conversion of lactate to pyruvate with the reduction of NAD to NADH which can be detected fluorescently (Decker and Lohmann-Matthes, 1988). LDH activity in the medium was assessed at 24 h of reperfusion and compared with maximum LDH release in medium after cells were frozen/thawed. The percentage death (% of LDH release) was calculated by dividing the experimental time point by the maximum LDH release values ×100. In parallel experiments propidium iodide (PI, 1 mg/ml) and Hoechst33258 (Sigma, 1 mg/ml) fluorescence were used to determine the percentage of cell death (Stary et al., 2017). Automated fluorescent image capture was performed at 200× using a Lumascope^TM^ 720 (Etaluma, Carlsbad, CA, United States) as we have previously performed (Xia et al., 2018). The number of PI-positive and Hoechst-positive cells were quantified using ImageJ software (v1.49b, National Institutes of Health, United States) and expressed as percentage of total cells.

### *In vivo* Transient Focal Cerebral Ischemia and Exosome Treatment

All experimental protocols using animals were performed according to protocols approved by the Stanford University Animal Care and Use Committee and in accordance with the NIH guide for the care and use of laboratory animals. Adult male CB57/B6 mice (*n* = 16, 25–30 g from Jackson Lab) were anesthetized with 2% isoflurane in balance O_2_ by facemask and focal cerebral ischemia was produced by 1 h of middle cerebral artery occlusion (MCAO) using a silicone-coated 6-monofilament (Doccol Co. Redlands, CA, United States) followed by reperfusion as we have previously performed (Stary et al., 2015, 2017). Rectal temperature was maintained at 37 ± 0.5°C controlled by a homeothermic blanket control unit (Harvard Apparatus, Holliston, MA, United States). Temperature and respiratory rate were monitored continuously. Mice were randomized to surgery, and mice with evidence of hemorrhage (*n* = 1 each group) were excluded from analysis and further treatment. Mice received treatment 2 h after reperfusion, a time point similar to when most stroke patients receive intervention (144 mins, (Saver et al., 2013)). Animals were re-anesthetized and either exosomes (10 ug total protein diluted in 100 μl saline, *n* = 7) or sterile saline alone (10 μl, *n* = 7) was administered into the surgically re-exposed internal jugular vein. This dose was chosen based on a prior study by Otero-Ortega et al. (2018) which administered 100 mg IV to rats, and scaled down to mice by body weight. Neurological status was assessed by neurologic deficit score 24 h after reperfusion (Xiong et al., 2011) using the following scoring system: 0 – no observable neurological deficits, 1 – failure to extend right forepaw; 2 – circling to the right; 3 – falling to the right; and 4 – cannot walk spontaneously. Cerebral infarction volume was determined after 4 days with 2,3,5-triphenyltetrazolium chloride (TTC, Sigma) staining of brains. Four sections/mouse were analyzed by a blinded observer and corrected for edema as described previously (Han et al., 2009) using ImageJ (v1.49b). Percent brain infarction was calculated as the area of the contralateral hemisphere minus the ipsilateral heather portion/contralateral hemisphere × 100, averaged for the sections.

### Statistical Analysis

**FIGURE 2:**
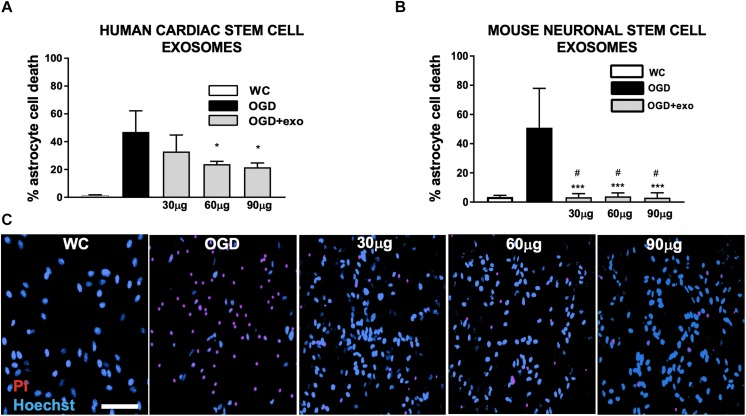
Protection of mouse primary astrocytes against ischemic injury with exosomes derived from human iCMs and exosomes derived from mouse NSCs. **(A)** Cell death assessed by lactate dehydrogenase (LDH) release from mouse primary astrocyte cultures after oxygen/glucose deprivation injury (OGD) with/without exosomes derived from human iCMs. **(B)** Cell death assessed by LDH in mouse primary astrocyte cultures after OGD with/without exosomes derived from mouse NSCs. **(C)** Examples of astrocyte cultures stained to assess cell death with propidium iodide (PI, red) after OGD and treated with varying concentrations of exosomes derived from mouse neuronal stem cells. Cells are counterstained with Hoechst (blue) which stains all nuclei. Data are expressed as mean ± SD, all graphs represent pooled data from three individual experiments (*n* = 4–6 per treatment group for each individual experiment). ^∗^*p* < 0.05, ^∗∗∗^*p* < 0.0005, compared with the control group. ^#^*p* < 0.05 versus same dose exosomes derived from human iCMs. WC = wash control.

**FIGURE 3:**
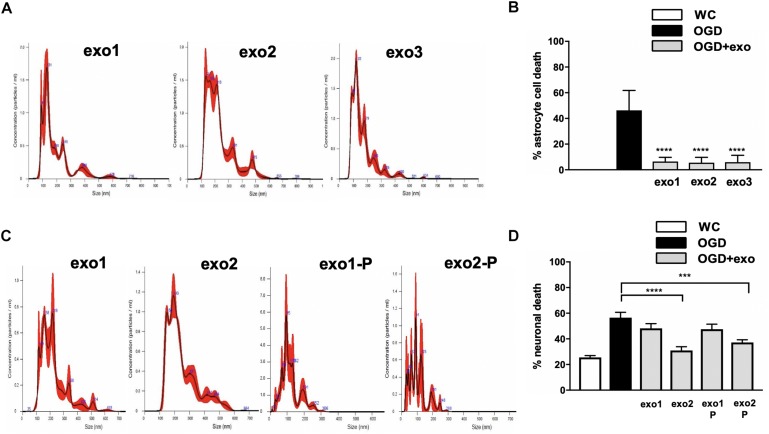
Protection with mouse neural stem cell (NSC)-derived exosome in primary mouse astrocyte cultures and mouse primary mouse neuronal cultures after ischemic injury. **(A)** Microparticle size distribution analysis of exosomes isolated from three separate mouse NSC cultures. **(B)** Astrocyte cell death with exosomes isolated from three separate mouse NSC cultures applied at 60 ug/well during oxygen/glucose deprivation (OGD), assessed by lactate dehydrogenase (LDH) release. **(C)** Microparticle size distribution analysis of exosomes derived from two separate mouse NSC cultures with standard purification (“exo1” and “exo2”) or an additional purification step (“exo1-P” and “exo2-P”) to remove residual polyethylene glycol (PEG) beads. **(D)** Neuronal cell death after OGD with exosomes derived from two separate mouse NSC cultures with either standard purification or an additional purification step applied at 60 ug/well. Data are expressed as mean ± SD, graphs represent pooled data from three individual experiments, (*n* = 4–6 per treatment group for each individual experiment). ^∗∗∗^*p* < 0.0005, ^∗∗∗∗^*p* < 0.0001. WC = wash control.

**FIGURE 4:**
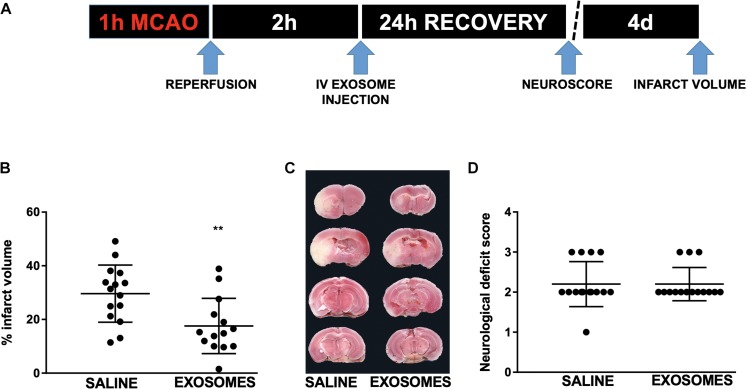
Effect of post-injury intravenous treatment with exosomes from mouse NSCs on *in vivo* injury from transient middle cerebral artery occlusion (MCAO). **(A)** Injury and treatment workflow. Mice were subjected to 1 h MCAO and treated after 2 h reperfusion with either intravenous (IV) saline or exosomes derived from mouse NSCs. **(B)** Quantification of infarct volume 4 days after MCAO in mice treated with either saline or mouse NSC-derived exosomes. **(C)** Representative TTC-stained brain sections with saline or exosome treatment (infarcted areas are lighter in color). **(D)** Neurological deficit 24 h following MCAO in mice treated with saline or NSC-derived exosomes. Data are expressed as mean ± SD, ^∗∗^*p* < 0.0046 versus saline (control) and exosome; *n* = 15–14/treatment group. TTC = 2,3,5-Triphenyltetrazolium chloride.

All values are presented as mean ± SD. For comparisons with more than two groups (i.e., Figures 2, 3), one-way ANOVA was used. For comparison of two groups (i.e., Figure 4), student’s *t*-test was used; Bonferroni *post-hoc* analysis was applied for statistical significance. GraphPad Prism 7.0 was used for statistical analysis and to plot graphs.

## Results

To investigate whether exosomes-derived from human or mouse stem cells had any effect on astrocytic cell death induced by OGD-reperfusion, we applied exosomes from either human iCMs or mouse NSC cultures immediately prior to and during OGD to confluent primary mouse astrocyte cultures. Both exosomes from human iCMs (Figure 2A) and exosomes from mouse NSCs (Figure 2B) exerted a neuroprotective effect. Exosomes derived from human iCMs reduced astrocytic LDH release from 47 to 32.9% at 30 ug/well, and then significantly (*p* < 0.05) reduced injury to 23.8% at 60 ug/well and to 21.6% in 90 ug/well. Comparatively, mouse NSC-derived exosomes treatment significantly (*p* < 0.0005) reduced LDH release from astrocyte cultures at all doses: from 50.9 to 3.34% with 30 ug/well, 3.9% with 60 ug/well and 3% with 90 ug/well. In comparison with the same dose human iCM-derived exosomes, mouse NSC-derived exosomes provided significantly (*p* < 0.05) greater protection against *in vitro* ischemia in these astrocyte cultures. Protection was confirmed in parallel *via* propidium iodide/Hoescht co-staining (Figure 2C).

To further validate these observations, we tested the protective effect of exosomes isolated from three additional culture dissections of mouse NSCs. Exosome quality and concentration from each dissection were first independently validated (Figure 3A) and were similarly significantly protective when applied at 60 ug/well during OGD, as assessed either by LDH release (Figure 3B: 46.32 to 6.5% for exosome 1, 5.53% for exosome 2 and 5.93% for exosome 3) or PI/Hoescht staining (data not shown).

Next, to assess whether protection by stem cell-derived exosomes is cell-type specific, we assessed their effect in primary neuronal cultures subjected to OGD. We observed that while NSC-derived exosomes were protective neuronal cultures were sensitive to trace amounts of the purification agent PEG 8000 and required an additional purification step (Figure 3C). Treatment with 60 ug/well NSC-derived exosomes during OGD reduced neuronal cell death significantly (*p* < 0.05) only when subjected to additional purification: from 56.58% to 30.97 for exo1-P, and to 37.18 by exo2-P (Figure 3D). We failed to observe any significant protection in primary neuronal cultures treated with human iCM-derived exosomes (data not shown).

Finally, to advance the clinical application of NSC-derived exosomes we assessed the effect of post-injury application in a mouse model of ischemic stroke. Mice were treated 2 h after reperfusion following 1 h of MCAO with an intravenous injection of NSC-derived exosomes (Figure 4A) or with saline alone. Relative to saline control, NSC-derived exosome treatment resulted in a significantly reduced infarct volume from 29.62 ± 2.755 to 17.59 ± 2.75% (*p* < 0.005; Figures 4B,C) 4 days after injury. However, we did not observe a significant difference in neurological deficit score (Figure 4D) at 24 h reperfusion.

## Discussion

In the present study, we first assessed *in vitro* the protective effect of mouse NSC- and human cardiac iCM-derived exosomes in primary mouse astrocyte cultures subjected to ischemic injury. Interestingly, we observed that application of human cardiac iCM-derived exosomes was protective in primary mouse astrocytes. These observations validate the clinical utility of utilizing rodent models of cerebral ischemia for pre-clinical testing human-derived exosomes, which will likely be available for clinical therapy in the near future, and also highlight that xenotransplantation of exosomes derived from other species may be a possible therapeutic route for human neuroprotection. However, comparatively, treatment with mouse NSC-derived exosomes resulted in near-perfect preservation of mouse astrocyte viability. Subsequently, we observed that mouse NSC-derived exosomes applied to mouse primary neuronal cultures also had a more modest (but significantly) protective effect against *in vitro* ischemia compared with the marked protection of NSC-derived exosomes applied to primary astrocyte cultures. Together these findings suggest that *in vivo* exosome-mediated neuroprotection may be primarily mediated by astrocytes in a species and/or organ-dependent manner.

Astrocytes play a key role in neuroprotection by maintaining cellular ion homeostasis and pH, sequestering excess neurotransmitter, and in neuronal synaptic signaling (Clarke and Barres, 2013; Liu et al., 2017). Astrocytes also mediate neuronal energy homeostasis by providing energy substrate and regulating neuronal mitochondrial biogenesis and degradation (Davis et al., 2014). Notably Hayakawa et al. (2016) recently demonstrated that astrocytes are capable of direct transfer of functional mitochondria to neurons, and that suppression of this process worsens ischemic injury. Relevant to the results from the present study, Xin et al. (2017) recently demonstrated that improved functional recovery after MCAO from *in vivo* treatment with exosomes derived from microRNA 133b-overexpressing mesenchymal stem cells was dependent on secondary exosome release from astrocytes. Both embryonic and adult neurogenesis is an astrocyte-mediated process (Sultan et al., 2015; Platel and Bordey, 2016), and specific to stroke, reactive astrocytes have been observed to behave as neural progenitor cells, an effect tied to latent activation of embryonic neurotrophic pathways in astrocytes (Gotz et al., 2015). Building on our observations from the present study of enhanced astrocyte protection with NSC-derived exosome, future investigations should assess *in vivo* whether NSC-derived exosome administration can recapitulate astrocyte-mediated neuro-restoration after stroke.

Exosomes are also central to embryonic intercellular communication in the developing nervous system and play a major role in brain development and function (Sharma et al., 2013). Exosomes released by glia regulate neuronal signaling and plasticity (Wang et al., 2012a; Fruhbeis et al., 2013; Gosselin et al., 2013) and astrocyte-derived exosomes have been demonstrated to promote neurite outgrowth and neuronal survival after oxidative stress (Wang et al., 2011). Xin et al. (2013a) originally demonstrated that systemic treatment of stroke with cell-free exosomes derived from mesenchymal stem cells significantly improved neurologic outcome and contributed to neurovascular remodeling. They noted that exosome-based therapies represented a safer therapeutic approach than cell-based therapies, as exogenously administered cells have the potential to generate emboli and to self-replicate (Wang et al., 2012b), and that protection and enhanced recovery of stem cell-based therapies are mediated by exosomal trafficking (Xin et al., 2012, 2013b). One aim of the present study was to demonstrate proof-of-principle whether clinical administration of exosomes derived from NSCs could minimize the evolution of injury from stroke. We observed that NSC-derived exosomes provided significant protection against ischemic stroke injury when given as post-MCAO IV therapy. Clinically, approximately 65–70% of stroke patients receive care within 3.5 h after onset of stroke symptoms to be treated with thrombolysis (Saver et al., 2013; Benjamin et al., 2019). The results from the present study with IV exosome treatment at 2 h post-stroke represents a realistic clinical scenario whereby exosomes could be co-administered as an adjuvant therapy with thrombolytics. Future investigations should extend these observations to assess the efficacy of direct (intracerebroventricular or intrathecal) NSC-derived exosome therapy on both acute and extended injury phases and clinical outcomes, with and without thrombolytic therapy.

One limitation of the present study was that we did not see improvement in gross motor function at 24 h post-MCAO with exosome treatment. This may be related to the short interval between post-injury exosome intervention (2 h) and our assessment of post-injury neuroscore (24 h), and/or the relative insensitivity of a 4-point test of gross motor function, although we have utilized this test to reliably demonstrate neuroprotection at 24 h in the past (Xiong et al., 2011; Xia et al., 2018). Another contributing factor may have been organ sequestration of circulating exosomes given IV or possibly secondary to inadequate dosing, however, in this mouse ischemic stroke model we employed a similar dosing regimen scaled to prior studies in rats (Xin et al., 2013b, 2017). Future studies would benefit from assessing long-term neurobehavioral outcome with repeated IV exosome dosing to more accurately assess the clinical potential of NSC-derived exosome therapy.

Another caveat is that we did not demonstrate the presence of the exosomes within the brain with IV treatment, leaving the possibility that protection from systemic exosome administration may have been mediated by systemic responses, possibly immune-mediated. Indeed, recent studies (Zagrean et al., 2018) indicate that altered immune system signaling in response to stroke may be communicated *via* exosome-mediated glial-neuronal crosstalk. Future studies should seek to develop methods to assess cell-type specific and sub-regional localization of exogenously applied exosomes which would provide additional insight into their therapeutic mechanism of action and expand their potential as a novel adjuvant stroke therapy.

Finally, in order to advance clinical relevance future pre-clinical studies should fully implement the stroke therapy academic industry roundtable (STAIR) and the stem cell therapies as an emerging paradigm in stroke (STEPS) recommendations (Lapchak et al., 2013). Combined, these recommendations include: randomized and blinded studies; efficacy in two or more laboratories; replication in a second species; consideration of age and gender; consideration of route of administration, and useful therapeutic window and dose response; defining inclusion/exclusion criteria; conducting full power analysis and sample size calculations; disclosing potential conflicts of interest; extended functional outcome (minimum of 1 month); and stratifying ischemic and hemorrhagic stroke subgroups. While the present study employed several of these criteria (randomization and blinding, clinical route of administration, etc.), future studies should assess exosome efficacy in females and in aged animals, with a focus on extended functional recovery.

## Data Availability

The datasets generated for this study are available on request to the corresponding author.

## Ethics Statement

All animal experiments were approved by Stanford University Animal Care and Use Committee (Stanford, CA, United States) and conducted according to the National Institutes of Health guidelines for animal welfare.

## Author Contributions

XS performed experiments and acquired the data. J-HJ prepared biological reagents and the manuscript. OA prepared the manuscript. MS prepared biological reagents. RG and PY assisted in experimental design and data analysis and provided funding. CS assisted in experimental design and data analysis, prepared the manuscript, and provided funding.

## Conflict of Interest Statement

The authors declare that the research was conducted in the absence of any commercial or financial relationships that could be construed as a potential conflict of interest.
